# Insecticide resistance exerts significant fitness costs in immature stages of *Anopheles gambiae* in western Kenya

**DOI:** 10.1186/s12936-021-03798-9

**Published:** 2021-06-09

**Authors:** Joyce K. Osoro, Maxwell G. Machani, Eric Ochomo, Christine Wanjala, Elizabeth Omukunda, Stephen Munga, Andrew K. Githeko, Guiyun Yan, Yaw A. Afrane

**Affiliations:** 1 Entomology Section, Centre for Global Health Research, Kenya Medical Research Institute, Kisumu, Kenya; 2 Department of Medical Laboratory Sciences, Masinde Muliro University of Science and Technology, Kakamega, Kenya; 3 Centre for Global Health Research, Kenya Medical Research Institute, Kisumu, Kenya; 4 Program in Public Health, College of Health Sciences, University of California, Irvine, CA 92697 USA; 5 Department of Medical Microbiology, University of Ghana Medical School, College of Health Sciences, University of Ghana, Accra, Ghana

**Keywords:** *Anopheles gambiae*, Insecticide resistance, Fitness, Larval life-traits

## Abstract

**Background:**

Despite increasing documentation of insecticide resistance in malaria vectors against public health insecticides in sub-Saharan Africa, there is a paucity of information on the potential fitness costs of pyrethroid resistance in malaria vectors, which is important in improving the current resistant management strategies. This study aimed to assess the fitness cost effects of insecticide resistance on the development and survival of immature *Anopheles gambiae* from western Kenya.

**Methods:**

Two-hour old, first instar larvae (L1) were introduced and raised in basins containing soil and rainwater in a semi-field set-up. Each day the number of surviving individuals per larval stage was counted and their stage of development were recorded until they emerged as adults. The larval life-history trait parameters measured include mean larval development time, daily survival and pupal emergence. Pyrethroid-resistant colony of *An. gambiae *sensu stricto and susceptible colony originating from the same site and with the same genetic background were used. Kisumu laboratory susceptible colony was used as a reference.

**Results:**

The resistant colony had a significantly longer larval development time through the developmental stages than the susceptible colony. The resistant colony took an average of 2 days longer to develop from first instar (L1) to fourth instar (L4) (8.8 ± 0.2 days) compared to the susceptible colony (6.6 ± 0.2 days). The development time from first instar to pupa formation was significantly longer by 3 days in the resistant colony (10.28 ± 0.3 days) than in susceptible colony (7.5 ± 0.2 days). The time from egg hatching to adult emergence was significantly longer for the resistant colony (12.1 ± 0.3 days) than the susceptible colony (9.6 ± 0.2 days). The pupation rate (80%; 95% (CI: 77.5–83.6) *vs* 83.5%; 95% (CI: 80.6–86.3)) and adult emergence rate (86.3% *vs* 92.8%) did not differ between the resistant and susceptible colonies, respectively. The sex ratio of the females to males for the resistant (1:1.2) and susceptible colonies (1:1.07) was significantly different.

**Conclusion:**

The study showed that pyrethroid resistance in *An. gambiae* had a fitness cost on their pre-imaginal development time and survival. Insecticide resistance delayed the development and reduced the survivorship of *An. gambiae* larvae. The study findings are important in understanding the fitness cost of insecticide resistance vectors that could contribute to shaping resistant management strategies.

**Supplementary Information:**

The online version contains supplementary material available at 10.1186/s12936-021-03798-9.

## Background

The development and spread of insecticide resistance threatens the control of vectors of infectious diseases in sub-Saharan Africa [1]. The continued use of insecticides for public health interventions and agricultural purposes seems to have generated high selective pressure on mosquito populations leading to the development of insecticide resistance in mosquito vectors [2–4]. Resistance to insecticides in malaria vectors has mainly been linked to the overexpression of detoxifying enzymes or enzyme structural changes that increase metabolic activity and target-site modification [5, 6]. This ability to resist insecticides through different mechanisms may present a fitness cost to resistant genotypes with negative effects in their development, reproductive aspects and vector competence which could affect the vectorial capacity of the malaria vectors [7].

Environmental selection pressure may select for certain phenotypes that will adapt to the new environment. It is hypothesized that phenotypic changes in an organism may have deleterious effects when the organism returns to its old environment [8]. For instance, resistant mosquito genotypes are believed to have an adaptive advantage in the insecticide environment resulting in increased resistance levels and this tends to decrease in the absence of insecticides suggesting the existence of a fitness cost [9]. The development and maintenance of resistant mechanisms in mosquitoes are thought to divert energy and resources associated with the primary physiological process, such as fecundity and longevity of individuals leading to a biological cost [8, 10]. Overexpression of metabolic enzymes and genes in resistant mosquitoes are thought to re-allocate primary energetic resources from other life-history traits, e.g., egg production and larval development to maintain secondary metabolic pathways involved in defence resulting in a fitness cost [11]. Changes in the insecticide target site may result in a fitness cost if the molecular alteration or the expressed genes are essential for the viability of the insect impairing the resistant individuals’ development and reproductive fitness [9]

Fitness costs associated with resistance have been reported to affect larval development and reproductive fitness of *Culex* and *Aedes* mosquitoes carrying resistances genes [9, 12–17]. Studies by Alout et al*.* [18] have also documented the fitness cost of insecticide resistance alleles on the vector competence of resistant phenotypes [18]. Currently, little is known about the effects of insecticide resistance mechanisms on the life-history parameters of *Anopheles gambiae *sensu stricto (*s.s.*) the major malaria vector in Africa. Few studies have been reported on the fitness cost of insecticide resistance on mosquito life-history traits, and many of these have utilized mosquito samples with different genetic backgrounds which could pose a challenge, as the life history traits could be influenced by other genetic factors beyond those related to insecticide resistance [19, 20]. This study investigated the fitness cost of insecticide resistance on the development and survival of immature *An. gambiae* from western Kenya using a pyrethroid-resistant population and susceptible populations originating from the same genetic background.

## Methods

### Mosquito population used in the study

Mosquito strains used in this study consisted of deltamethrin-selected resistant colony (hereafter referred to as resistant colony) and a no-insecticide-exposed susceptible colony (hereafter referred to as susceptible colony) that were collected from Bungoma in western Kenya.

### Resistant colony

Briefly, the resistant colony was selected using 0.05% deltamethrin at every generation. The 6^th^ generation of this colony was used in this study and had a mortality rate of 20%. The two *kdr* mutations L1014S (77%) and L1014F (23%) were present, with high frequencies of L1014F compared to the parent population (0.09). Also, resistance in this colony was mainly mediated by cytochrome P450 detoxification enzyme [21]

### Susceptible colony

This colony shared the same genetic background as the resistant colony but was raised in the absence of insecticide selection pressure [21]. The 13th generation of this colony was used for this study. At the 13^th^ generation, the colony was showing 97.3% mortality when exposed to 0.05% deltamethrin (Additional file 1: Fig. S1). Only L1014S was detected in this colony as it was already fixed in the parent population (0.88) and was depicted to play little role in pyrethroid resistance [21].

The resistant and susceptible colony differed in cytochrome p450 enzyme activities and L1014F frequencies but not in L1014S. Pre-exposure of the resistant mosquitoes to synergist piperonyl butoxide restored susceptibility of these mosquitoes to pyrethroids, confirming the role of monooxygenase enzyme in the observed pyrethroid resistance[21].

### Kisumu strain

The *An. gambiae* Kisumu reference laboratory strain, which has been colonized since 1954 and is free of any detectable insecticide resistance mechanism, was used as a control susceptible strain in all bioassays.

The mosquito colonies were maintained in three (3) lineages in the insectary at the Centre for Global Health Research (CGHR), Kenya Medical Research Institute (KEMRI) in Kisumu, under standard conditions (25 ± 2 °C; 80% ± 4% relative humidity with a 12 h: 12 h light/dark cycle). Larvae were fed on tetramin baby fish food and brewer’s yeast daily and adults maintained in a 10% sugar solution. All three lineages were used for the experiment, with each lineage being used as a replicate.

### Life table experiments

Three parameters were evaluated to examine the fitness cost: mean larval development time (L1-Pupal), pupal emergence and daily survival. The parameters were measured under semi-field conditions after every 24 h and focused on the difference between mosquitoes expressing different levels of insecticide resistance originating from the same genetic background. The Kisumu susceptible laboratory strain was used as a control.

### Experimental design

A total of 27 semi-natural habitats (9 replicates per colony) were created using plastic washbasins (35 cm in diameter and 15 cm deep) at CGHR/KEMRI/ compound in Kisumu, according to the method described by Afrane et al*.* [22]. Two kg of soil from breeding sites and 5 l of rainwater were added to each washbasin. Two holes (3 cm in diameter) were created near the top edge of each washbasin to maintain a constant water level when it rained. The holes were covered with a screen (mesh size 200 μm) to prevent larvae from being washed away[22, 23]. Thirty 2-h old larvae from the three lineages of these colonies, as a replicate were placed separately in different basins. Each washbasin was covered with a nylon netting to prevent predators and wild mosquitoes from ovipositing eggs in the washbasin. The surviving larvae in each washbasin were checked and counted daily and their numbers were recorded. The stage of development of individual larvae was also identified using the identification keys of Gilles and Coetzee [24] and recorded to measure the development time per each larval instar. Pupae were picked, recorded and transferred to pupal cups, which were then placed in cages for adult emergence. Pupae were monitored daily and the number and sex of emerging adults recorded. All larvae from the three colonies were reared through adults in semi-field conditions. The mean length of time from the first instar to adult emergence for each sex, as well as the ratio of male to female emergences was recorded for each colony. The experiment was repeated four times.

### Data analysis

Mean larval development time was defined as the average time of the first instar larvae to develop into adults. Mean pupation time was calculated as the average time taken for the first instar larva to pupate. The male and female development time was recorded differently because they take different times to emerge. The pupation rate was calculated as the percentage of the first instar larvae that emerged to pupae. The emergence rate was calculated as the percentage of the pupae that emerged to adults. Analysis of variance (ANOVA) was conducted to determine the effects of insecticide resistance on the pupation time, larval development time, pupation rate and emergence rate of the resistant colony, susceptible colony and the Kisumu reference *An. gambiae s.s*. Tukey HSD post hoc tests were used to determine the statistical significance of the difference in larval development time, pupation rate and emergence rate among the resistant, susceptible and the Kisumu reference colonies. Kaplan–Meier survival test was used in the testing for differences in larval survivorship among the resistant, susceptible and the Kisumu reference mosquitoes. The level of significance was set at 0.05 for all tests.

## Results

### Effect of insecticide resistance on larval development

The mean development time from first instar (L1) to second instar (L2) for the resistant colony was 4.9 ± 0.2, while the susceptible colony was 3.4 ± 0.1 and 3.4 ± 0.1 for the Kisumu strain (F_2,63_ = 44.43, P < 0.0001; Table 1). The average length of larval development time (L1-L2) for the resistant colony was 1.5 days longer compared to the susceptible colony. The time for resistant colony to develop from first instar (L1) to third instar (L3) was 6.9 ± 0.2 days while the susceptible colony was 4.9 ± 0.2 and 4.8 ± 0.2 days for the Kisumu colony. The development time (L1-L3) for the resistant colony was 2 days longer compared to the susceptible colony (F_2,63_ = 44.61_,_ P < 0.0001). The mean pre-imaginal development time from first instar (L_1_) to fourth instar (L_4_) of the resistant colony was 8.8 ± 0.2, while the susceptible colony was 6.6 ± 0.2 and 6.3 ± 0.2 for Kisumu laboratory susceptible mosquitoes. The resistant colony took a significantly longer period (2.2 days) to develop from L_1_-L_4_ with respect to the susceptible colony (F_2,63_ = 47.06, P < 0.0001).

**Table 1:** Comparison of larval instar development time among the resistant, susceptible and Kisumu colonies

Population	Larval instar development time (days)		
	L2^i^	L3^ii^	L4^iii^
	Mean ± SE^*^	Mean ± SE^*^	Mean ± SE^*^
Resistant colony	4.9 ± 0.2^b^	6.9 ± 0.2^b^	8.8 ± 0.2^b^
Susceptible colony	3.4 ± 0.1^a^	4.9 ± 0.2^a^	6.6 ± 0.2^a^
Kisumu strain	3.4 ± 0.1^a^	4.8 ± 0.2^a^	6.3 ± 0.2^a^

^*^Values indicate mean and the standard error. The same superscript letters in each row indicate no significant difference (p > 0.05, ANOVA, followed by Tukey (HSD) test. ^i^ Duration of L1 to develop to the second instar larvae (L2). ^ii^ Duration of L1 to develop into the third instar larvae (L3). ^iii^ Duration of L1 to develop into the fourth instar larvae (L4)

### Pupation and emergence times between the resistant and susceptible colonies

The resistant colony reached pupal stage 10.28 ± 0.3 days after hatching as L1, whilst the susceptible colony took 7.5 ± 0.2 days. Development time from L1 to pupal stage was significantly longer in the resistant colony than in the susceptible colony (F_2,63_ = 39.45, P < 0.0001, Table 2). The Kisumu strain took 7.9 ± 0.2 days to pupate.

**Table 2:** Comparison of larval-life trait parameters of the resistant, susceptible and Kisumu strain of *Anopheles gambiae *s.s

Population	Pupation time (days)^1^	Pupation rate (%)^2^	Mean development time of Male (days)^3*^	Mean development time of females (days)^4*^	Emergence rate (%)^5^
Resistant colony	10.28 ± 0.3^b^	80 ± 0.03^a^	11.9 ± 0.30^b^	12.1 ± 0.3^b^	86.3 ± 0.04^a^
Susceptible colony	7.5 ± 0.2^a^	83.5 ± 0.03^a^	9.2 ± 0.2^a^	9.6 ± 0.2^a^	92.8 ± 0.02^a^
Kisumu strain	7.9 ± 0.2^a^	84.5 ± 0.03^a^	9.4 ± 0.2^a^	9.8 ± 0.2^a^	85.7 ± 0.02^a^

^*^Values indicate mean and the standard error. The same superscript letters in each row indicate no significant difference (p > 0.05, ANOVA, followed by Tukey (HSD) test. ^1^ Duration of L1 larvae developing to pupae.^2^ Per cent of larvae developing to pupae. ^3^ Duration of L1 larvae to develop to males. ^4^ Duration of L1 to develop into females. ^4^. ^5^ Percent of pupae developing to adults

The pupation rate in the resistant colony was 80% (95% CI: 77.5–83.6), while it was 83.5% (95% CI: 80.6–86.3) for the susceptible colony. Although the resistant colony took a longer time to develop, there was no significant difference in the pupation rate between the resistant and susceptible colonies (F_2,63_ = 0.084_,_ P > 0.05).

The proportion of pupae emerging to adults was high in the susceptible colony 92.8% compared to the resistant colony 86.3%. However, this was not statistically significant (F_2,63=_7.18, P > 0.05). The emergence rate for the Kisumu strain was 85.7% (Table 2). The emergence time for males and females in the resistant colony was 11.9 ± 0.3 and 12.1 ± 0.3, respectively, while the susceptible colony was 9.2 ± 0.2 and 9.6 ± 0.2 days, respectively. The male emergence time for the Kisumu strain was 9.4 days and 9.8 days for females. There was a significant difference between the emergence time for males and females in the resistant colony compared to the susceptible colony (males; F_2,63_ = 38.4, P < 0.05; females, F_2,63_ = 35.81, P < 0.05, Table 2).

The proportion of males emerged from the resistant and susceptible colonies was higher (resistant colony: 54.8% (95% CI: 50.4–59.3); susceptible colony: 54.5% (95% CI: 47.2–56.2) compared to the emerged females (resistant colony: 45.2% (95% CI: 40.7–49.6); susceptible colony: 45.5% (95% CI: 41.2–50) (Table 3). The sex ratio of females to males was significantly different for resistant colony 1: 1.21 (t = 2.5248, df = 42, P < 0.0154) and susceptible colony 1: 1.19 (t = 2.2525, df = 42, P < 0.029). Although the proportions of males to females was high in the Kisumu strain (51.7 *vs* 48.3%), this was not statistically significant (t = 0.854, df = 42, P > 0.05).

**Table 3:** The average number of male and female adults emerged from the three colonies

Colony	Sample size	Female^*^	Male^*^	Sex ratio
		Mean (%) ± SD^i^	Mean (%) ± SD^ii^	Female: Male^iii^
Resistant colony	485	45.2 ± 0.6	54.8 ± 0.6	1:1.2^a^
Susceptible colony	512	45.5 ± 0.5	54.5 ± 0.7	1:1.19^a^
Kisumu strain	472	48.3 ± 0.6	51.7 ± 0.6	1:1.07^b^

^*^Values indicate mean and the standard error. The same superscript letters in the last row indicate no significant difference. ^i^ Proportion of females that emerged. ^ii^ Proportion of males that emerged. ^iii^ Sex ratio of females to males

### Survivorship among the resistant and susceptible colonies

**Fig. 1:**
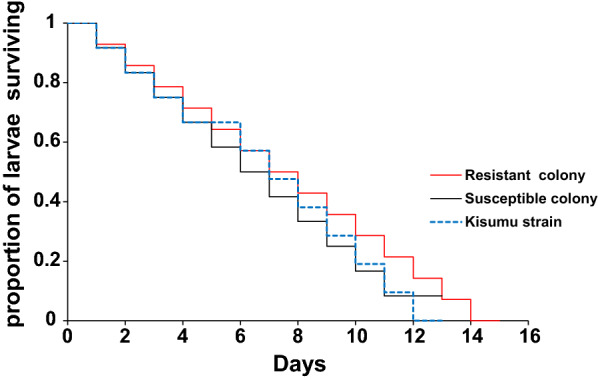
Larval survival probability of pyrethroid-selected resistant and susceptible colonies of *Anopheles gambiae *s.s

The resistant colony showed a longer survival time of 15 days, with a median survival length of 8 days compared to the susceptible colony that survived for 12 days with a median survival length of 6 days (Fig. 1). The Kisumu strain exhibited a very similar trend as the susceptible colony. When comparing the survival curves using Wilcoxon proportional hazard ratio test, there was no significant difference in larval survivorship between resistant and susceptible colonies (P = 0.43). The larval mortality rate was high in the resistant colony 20% (95% CI: 16.4–22.5), while in the susceptible colony it was 16% (95% CI: 13.5–19.2) and Kisumu strain was 17% (95% CI: 13.7–19.3). Although the mortality rate was high in the resistant colony, no significant difference was found between the colonies (F_2,39_ = 0.141, P > 0.05).

## Discussion

Under an evolutionary perspective, it is hypothesized that genetic changes arising as a result of insecticides' selective pressure can present a fitness cost to resistant insects bearing negative effects on their biological traits[25]. The study assessed larval development time and survivorship of *An. gambiae* colonies, exhibiting different insecticide resistance status. The results of this study demonstrate the existence of fitness cost in *An. gambiae s.s*. immature stages associated with pyrethroid resistance. Overall larval development time and survival was compromised in the resistant colony compared to the susceptible colony originating from the same background. The development time of the susceptible colony was remarkably similar to that of the susceptible Kisumu reference strain.

The study observed prolonged development time from one larval instar to the other in the resistant colony when compared with the susceptible colony whose development time was similar to the Kisumu strain. The majority of individuals from the susceptible colony and the Kisumu strain reached the pupal stage about 7 days after the hatching of the first instar, whereas the resistant colony took additional 3 days before pupation. These findings present an adaptive disadvantage on the resistant individuals as the amount of time spent in the natural breeding habitats in the field may impact their survival rates due to exposure to natural predations. They are also likely to suffer temporary or permanent loss of habitats before emerging to adults, which may, in turn, have a direct consequence on the vectorial capacity [7]. Similarly, studies on pyrethroid-resistant *Anopheles funestus*, *Culex quinquefasciatus, Aedes aegypti*, and *Aedes albopictus* have observed longer phase of larval development, unlike their susceptible counterparts [12, 14–16, 26].

Larval survivorship of the resistant colony was low, characterized by low pupation rates, high pupae mortality and decreased adult emergence compared to the susceptible and Kisumu colonies. These could be possibly due to the accumulation of harmful effects of genes related to insecticide detoxifying enzymes or molecular alterations on the target (*kdr* mutations). The success in survivorship of the susceptible colony could be attributed to the loss of resistance in them that could enable them to focus most of their energy on growth enhancement metabolic processes. The low larval survivorship in the resistant colony may present low vector population densities disabling effective malaria transmission by resistant mosquitoes. Similar studies have reported the negative effects associated with insecticide resistance on the biological characteristics of pyrethroid-resistant *Ae. albopictus* and *Culex pipiens* compared to their susceptible counterparts [17, 27]

It is important to highlight that monooxygenase enzyme was majorly implicated in the pyrethroid resistance of the selected colony even though *kdr* mutations were observed at high frequencies [21]. It is likely that the overproduction of monooxygenase would have committed resources important for primary biological functions, such as development to maintaining secondary functions, i.e., insecticide detoxification [25]. For instance, some studies have linked the staggered larval development time of resistant individuals with spending more time in the accumulation of nutrients to achieve the development threshold that triggers growth to the next stage as most of the resources are used to maintain resistance [28]. The findings of this study are similar to reports on *An. funestus* from West Africa harbouring 119F-GSTe2 resistant alleles which exhibited delayed larval development compared to the population without the resistant alleles [26]. The *kdr* mutation has been associated with a delay in the larval development of *Ae. aegypti* [9, 29]. The observed negative effects associated with insecticide resistance may affect the spread of insecticide resistance genes in a population, as the resistant individuals are likely to take a longer time to develop and emerge as adults, unlike the susceptible ones. Based on this, resistance management tactics may rely on this reduced fitness disadvantage to design integrated vector control management strategies with an aim of limiting the spread of insecticide resistance and maintaining the effectiveness of the existing vector control tools.

## Conclusion

This study revealed that there was a fitness cost associated with pyrethroid resistance in *An. gambiae*. Pyrethroid resistance resulted in fitness disadvantages as exhibited by the resistant colony that recorded slow larval development time and reduced survivorship. These negative fitness aspects associated with pyrethroid resistance could be possibly due to the accumulation of harmful or deleterious effects of genes related to monooxygenase detoxification enzyme and the co-occurrence of both L1014S and L1014F mutations in the resistant colony. These findings could be useful in developing better insecticide management strategies.

## Supplementary Information

**Additional file 1: Fig. S1.** Percentage mortality rates of the selected pyrethroid resistant *Anopheles gambiae* and unselected susceptible colonies. Mortality rate was measured using the WHO insecticide susceptibly tube bioassay for deltamethrin. Error bars indicate 95% confidence intervals. The 90% mortality threshold for declaring suspected resistance and 98% mortality threshold for calling full susceptibility based on the WHO criteria are indicated.

## Data Availability

The dataset supporting the conclusions of this article is included within the article.

## References

[1] Ranson H, Lissenden N (2016). Insecticide resistance in African Anopheles mosquitoes: a worsening situation that needs urgent action to mantain malaria control. Trends Parasitol.

[2] Diabate A, Baldet T, Chandre F, Akogbeto M, Guiguemde TR, Darriet F (2002). The role of agricultural use of insecticides in resistance to pyrethroids in *Anopheles gambiae* s.l. in Burkina Faso. Am J Trop Med Hyg..

[3] Stump AD, Atieli F, Vulule J, Besansky NJ (2004). Dynamics of the pyrethroid knockdown resistance allele in Western Kenyan populations of *Anopheles gambiae* in response to insecticide-treated bed net trials. Am J Trop Med Hyg.

[4] Mathias D, Ochomo E, Atieli F, Ombok M, Bayoh N, Olang G (2011). Spatial and temporal variation in the kdr allele L1014S in *Anopheles gambiae* s.s. and phenotypic variability in susceptibility to insecticides in Western Kenya. Malar J..

[5] Ranson H, N’guessan R, Lines J, Moiroux N, Nkuni Z, Corbel V (2011). Pyrethroid resistance in African anopheline mosquitoes: what are the implications for malaria control?. Trends Parasitol..

[6] Hemingway J, Hawkes NJ, McCarrol L, Ranson H (2004). The molecular basis of insecticide resistance in mosquitoes. Insect Biochem Mol Biol.

[7] Rivero A, Vezilier J, Weill M, Read AF, Gandon S (2010). Insecticide control of vector-borne diseases: when is insecticide resistance a problem?. PLoS Pathog..

[8] Coustau C, Chevillon C (2000). Resistance to xenobiotics and parasites: can we count the cost?. Trends Ecol Evol.

[9] Brito LP, Linss JG, Lima-Camara TN, Belinato TA, Peixoto AA, Lima JBP (2013). Assessing the effects of *Aedes aegypti kdr* mutations on pyrethroid resistance and its fitness cost. PLoS One..

[10] Alout H, Roche B, Dabiré RK, Cohuet A (2017). Consequences of insecticide resistance on malaria transmission. PLoS Pathog..

[11] Chevillon C, Raymond M, Guillemaud T, Lenormand T, Pasteur N (1999). Population genetics of insecticide resistance in the mosquito *Culex pipiens*. Biol J Linn Soc.

[12] Martins AJ, Bellinato DF, Peixoto AA, Valle D, Lima JBP (2012). Effect of insecticide resistance on development, longevity and reproduction of field or laboratory selected *Aedes aegypti* populations. PLoS One..

[13] Mebrahtu YB, Norem J, Taylor M (1997). Inheritance of larval resistance to permethrin in *Aedes aegypti* and association with sex ratio distortion and life history variation. Am J Trop Med Hyg.

[14] Hardstone MC, Lazzaro BP, Scott JG (2009). The effect of three environmental conditions on the fitness of cytochrome P450 monooxygenase-mediated permethrin resistance in *Culex pipiens quinquefasciatu*s. BMC Evol Biol.

[15] Li X, Ma L, Sun L, Zhu C (2002). Biotic characteristics in the deltamethrin-susceptible and resistant strains of *Culex pipiens pallens* (Diptera: Culicidae) in China. Appl Entomol Zool.

[16] Jaramillo-O N, Fonseca-Gonzalez I, Chaverra-Rodríguez D (2014). Geometric morphometrics of nine field isolates of *Aedes aegypti* with different resistance levels to lambda-cyhalothrin and relative fitness of one artificially selected for resistance. PLoS One..

[17] Chan HH, Zairi J (2013). Permethrin resistance in *Aedes albopictus* (Diptera: Culicidae) and associated fitness costs. J Med Entomol.

[18] Alout H, Ndam NT, Sandeu MM, Djegbe I, Chandre F, Dabiré RK (2013). Insecticide resistance alleles affect vector competence of *Anopheles gambiae* s.s. for *Plasmodium falciparum* field isolates. PLoS One..

[19] Leisnham PT, Sala L, Juliano SA (2014). Geographic variation in adult survival and reproductive tactics of the mosquito *Aedes albopictus*. J Med Entomol.

[20] Nkahe DL, Kopya E, Djiappi-Tchamen B, Toussile W, Sonhafouo-Chiana N, Kekeunou S (2020). Fitness cost of insecticide resistance on the life-traits of a *Anopheles coluzzii* population from the city of Yaoundé Cameroon. Wellcome Open Res.

[21] Machani MG, Ochomo E, Zhong D, Zhou G, Wang X, Githeko AK (2020). Phenotypic, genotypic and biochemical changes during pyrethroid resistance selection in *Anopheles gambiae* mosquitoes. Sci Rep.

[22] Afrane YA, Zhou G, Lawson BW, Githeko AK, Yan G (2007). Life-table analysis of *Anopheles arabiensis* in western Kenya highlands: effects of land covers on larval and adult survivorship. Am J Trop Med Hyg.

[23] Minakawa N, Omukunda E, Zhou G, Githeko A, Yan G (2006). Malaria vector productivity in relation to the highland environment in Kenya. Am J Trop Med Hyg.

[24] Gillies MT, De Meillon B (1968). The Anophelinae of Africa south of the Sahara (Ethiopian zoogeographical region).

[25] Kliot A, Ghanim M (2012). Fitness costs associated with insecticide resistance. Pest Manag Sci.

[26] Tchouakui M, Riveron JM, Djonabaye D, Tchapga W, Irving H, Soh Takam P (2018). Fitness costs of the glutathione S-transferase epsilon 2 (L119F-GSTe2) mediated metabolic resistance to insecticides in the major African malaria vector *Anopheles funestus*. Genes.

[27] Gazave É, Chevillon C, Lenormand T, Marquine M, Raymond M (2001). Dissecting the cost of insec*ticide resistance genes during the overwintering period of the mosquito* Culex pipiens. Heredity (Edinb).

[28] Diniz DFA, de Melo-Santos MAV, de Mendonça Santos EM, Beserra EB, Helvecio E, de Carvalho-Leandro D (2015). Fitness cost in field and laboratory *Aedes aegypti* populations associated with resistance to the insecticide temephos. Parasit Vectors.

[29] de Lourdes Macoris M, Martins AJ, Andrighetti MTM, Lima JBP, Valle D (2018). Pyrethroid resistance persists after ten years without usage against *Aedes aegypti* in governmental campaigns: Lessons from São Paulo State Brazil. PLoS Negl Trop Dis..

