# Supplementary figures and images for: Insecticide resistance exerts significant fitness costs in immature stages of Anopheles gambiae in western Kenya
Source: Malar J. 2021 Jun 9;20:259. doi: 10.1186/s12936-021-03798-9 (PMC8188659; doi:10.1186/s12936-021-03798-9)

## Slide 1
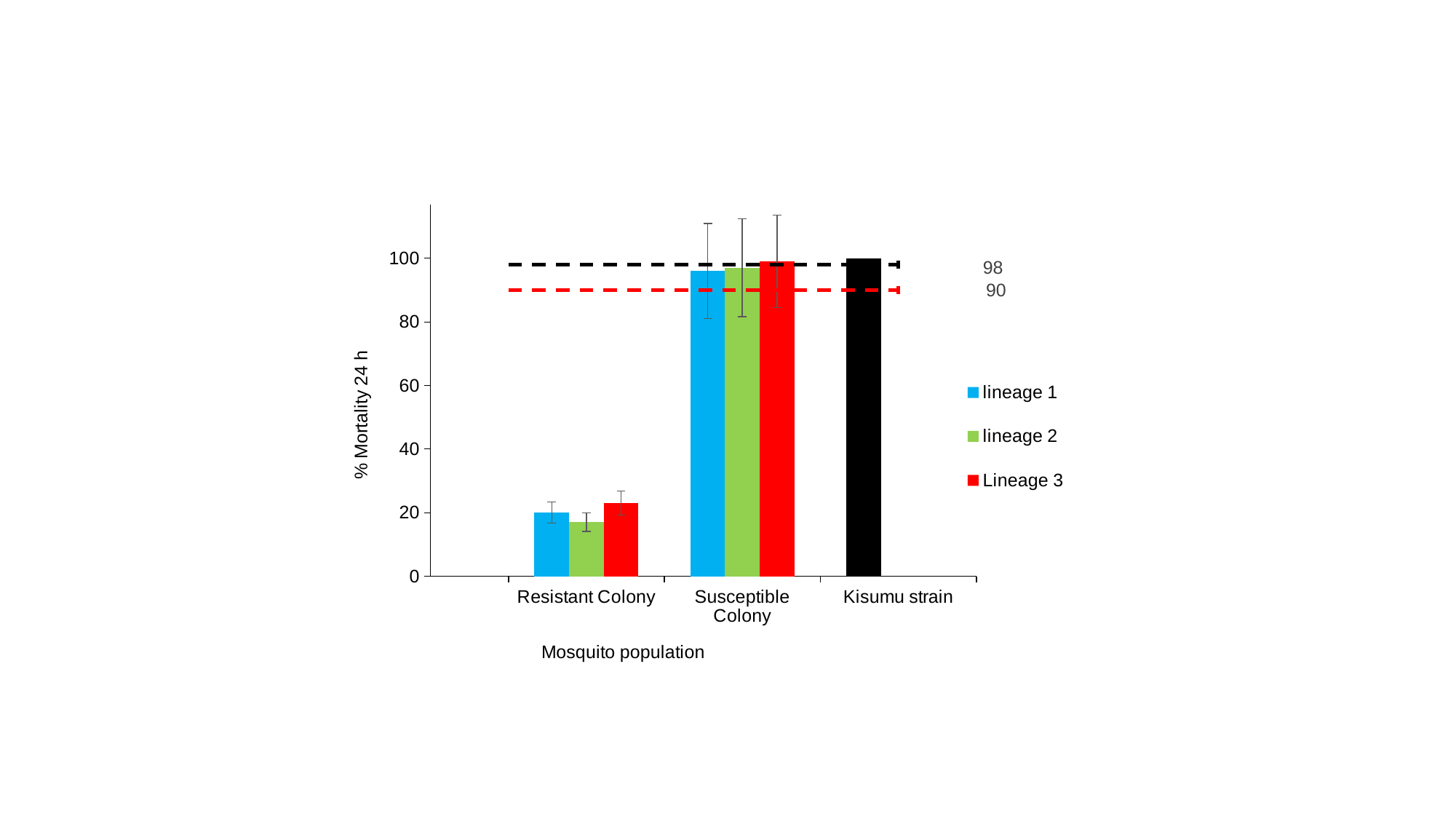

[unsupported chart]

Supplement: Supplementary file 1 — Additional file 1: Fig. S1. Percentage mortality rates of the selected pyrethroid resistant Anopheles gambiae and unselected susceptible colonies. Mortality rate was measured using the WHO insecticide susceptibly tube bioassay for deltamethrin. Error bars indicate 95% confidence intervals. The 90% mortality threshold for declaring suspected resistance and 98% mortality threshold for calling full susceptibility based on the WHO criteria are indicated. [file 12936_2021_3798_MOESM1_ESM.pptx]
